# A Disguising Fast-Growing Metachronous Melanoma and COVID-19

**DOI:** 10.7759/cureus.36108

**Published:** 2023-03-13

**Authors:** Alina Avram, Lucian G Scurtu, Mariana Costache, Olga Simionescu

**Affiliations:** 1 Department of Dermatology I, Colentina Clinical Hospital, Carol Davila University of Medicine and Pharmacy, Bucharest, ROU; 2 Department of Pathology, Bucharest University Emergency Hospital, Carol Davila University of Medicine and Pharmacy, Bucharest, ROU

**Keywords:** malignant melanoma, sars-cov-2, covid-19, nodular melanoma, skin cancer, multiple melanomas

## Abstract

An unusual case of a 52-year-old female with two metachronous melanomas is presented. An atypical fast-growing nodular melanoma appeared 18 months after the complete excision of an in situ melanoma and one month afterward a SARS-CoV-2 infection. Intra-nodal melanocytic proliferations were identified during lymph node assessment, raising important diagnostic and prognostic concerns. No melanoma susceptibility genes were found. This case report raises the question about the COVID-19 immunosuppression effect on the tumor microenvironment and the oncogenic potential of SARS-CoV-2. It also highlights the importance of clinical follow-up in melanoma patients, which was significantly delayed during the COVID-19 pandemic.

## Introduction

Melanoma represents a malignant tumor that develops from melanocytes and predominantly involves the skin. Its incidence has been increasing globally, especially in fair-skinned persons. In situ melanoma is a noninvasive subtype confined within the epidermis. Nodular melanoma represents 16% of invasive melanoma subtypes and usually presents an abrupt vertical growth phase with increased tumor thickness (Breslow index) and a less prominent epidermal lateral component [1]. The prevalence of multiple primary melanomas ranges in different studies from 1.3% to 8% [2,3]. Among patients who develop a second melanoma, over 70% develop melanoma within the first five years of the first malignancy and over 90% within the first 10 years [4].

The term "synchronous" refers to two (or more) independent primary tumors that appear within six months or less apart; asynchronous or metachronous melanomas usually arise more than six months apart [5]. The COVID-19 pandemic has had a catastrophic impact on melanoma morbidity and mortality due to delayed diagnosis and treatment. The tumor thickness and mitotic rate have increased during the pandemic [6]. This paper describes the case of a 52-year-old female who developed metachronous nodular melanoma one month after COVID-19.

## Case presentation

**Figure 1:**
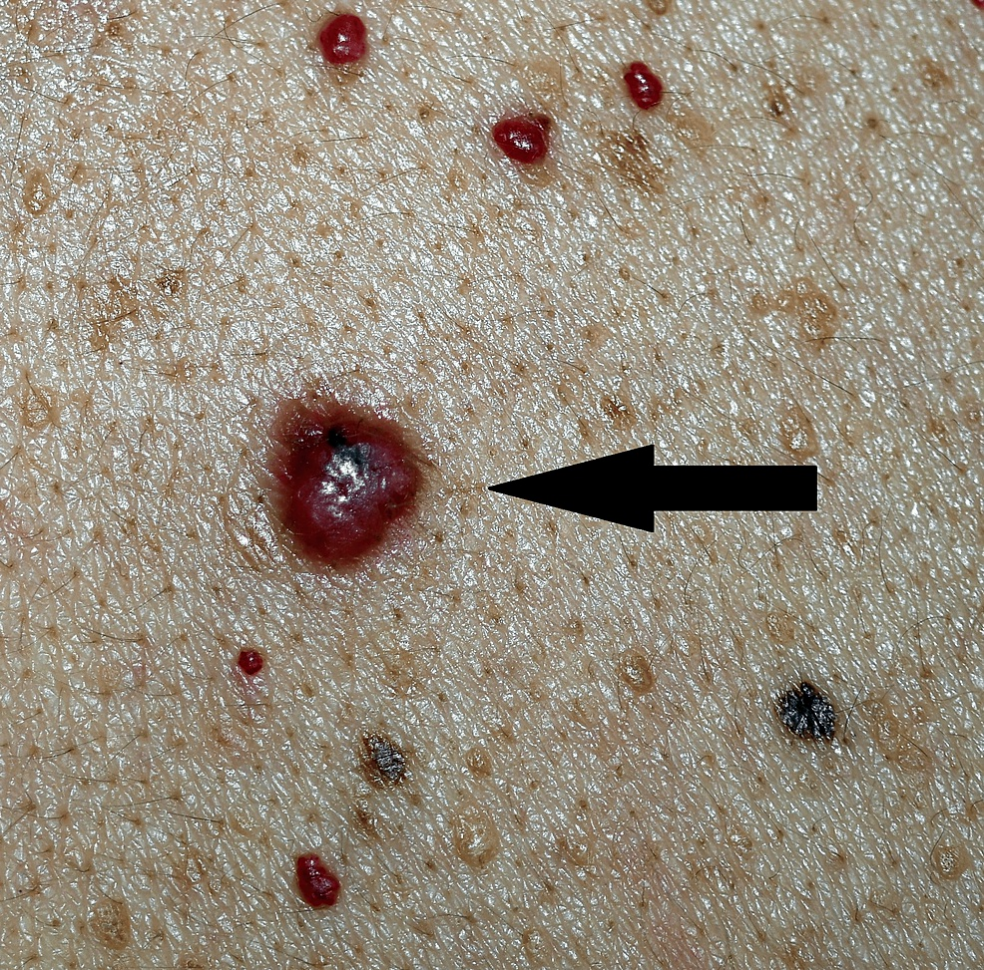
A 7 mm, smooth-surfaced, solitary, red nodule (arrow) localized on the posterior thorax, with a visible blue central globule.

A 52-year-old Caucasian female, with Fitzpatrick II skin type, atypical mole syndrome, and a history of in situ melanoma, completely excised in November 2019, had been followed up every six months with a total body skin examination. The patient had no family history of skin cancer and no personal history of excessive sun exposure, sunburns in childhood, or outdoor hobbies. This patient was an indoor worker, with intermittent sun exposure previous to her first melanoma. The patient denied sun exposure after the melanoma excision. In April 2021, the patient worried about a new, solitary, erythematous, dome-shaped, painless nodule located on the posterior thorax. This tumor (Figure 1) appeared one month apart from a SARS-CoV-2 infection and only two months after her last dermatology checkup. The patient did not require medical supervision for COVID-19 and had a favorable clinical outcome.

**Figure 2:**
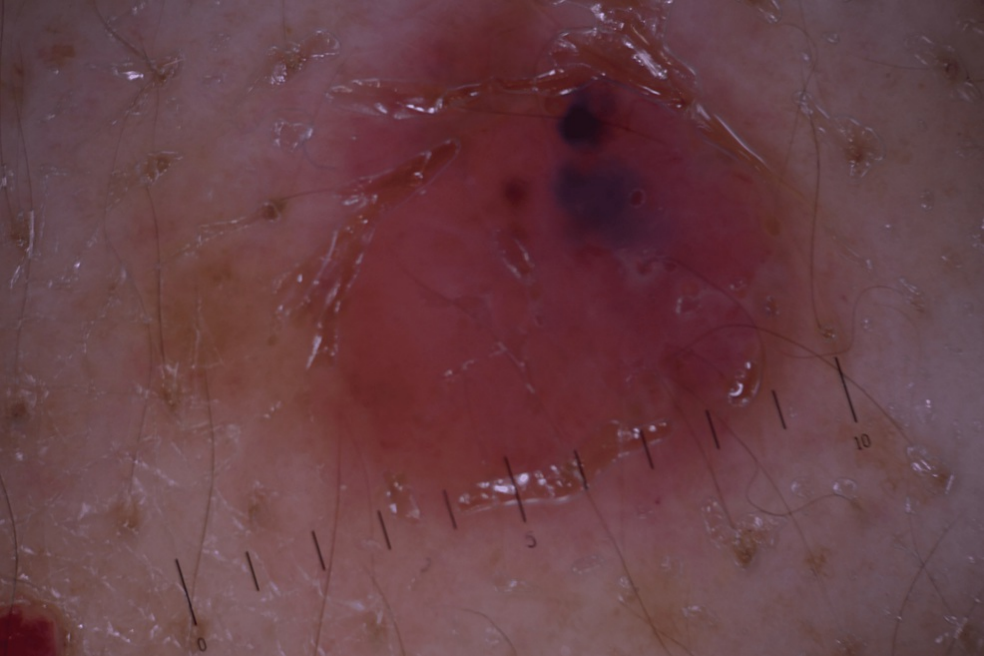
Digital dermoscopy reveals a nodular, red tumor, with large, blue-gray, ovoid structures.

The digital dermoscopy evaluation (Heine Delta 20 T {Heine Optotechnik, Gilching, Germany} attached to a Nikon digital camera {Nikon Corporation, Tokyo, Japan}) revealed the absence of criteria for melanocytic lesion and the presence of blue-gray ovoid structures, suggestive of basal cell carcinoma (Figure 2). The patient's complete blood count and urinalysis showed no anomalies. Given the melanoma history and the florid atypical mole syndrome phenotype, a standard 5 mm-margin scalpel excision was performed under local anesthesia and sedation.

**Figure 3:**
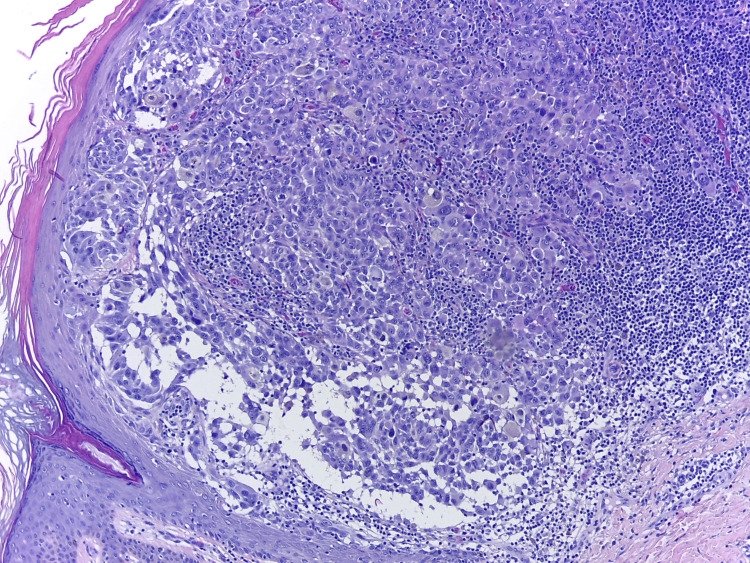
Nodular melanoma 1.47 mm, vertical growth phase. A densely cellular melanocytic tumor, consisting of round/oval cells and rare, elongated, bulky cells. Tumor cells present pleiomorphic, atypical, hyperchromatic nuclei, with rare mitoses and brown pigment within the cytoplasm. They are organized in nests at the dermo-epidermal junction or in islands within the dermis. Predominantly, lymphocytic infiltrates are present. Hematoxylin and eosin, original magnification: ×100.

**Figure 4:**
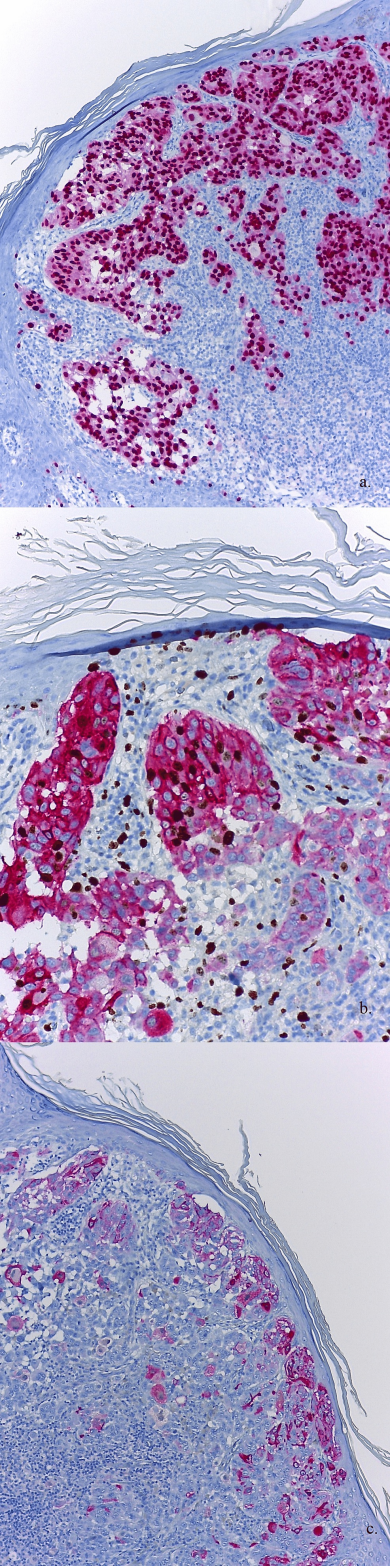
a) Nodular melanoma, vertical growth phase without radial growth phase: high nuclear immunostaining, anti-SOX-10. b) A double staining for Ki67 (DAB) positive in tumor nuclei and Melan-A/MART1 (Fast Red) positive within the cytoplasm. c) Heterogenous cytoplasmic immunostaining, anti-HMB-45. Original magnification: ×100. DAB, diaminobenzidine; SOX-10, SRY-related HMg-Box gene 10; HMB-45, human melanoma black 45; MART1, melanoma-associated antigen recognized by T cells 1

The path report revealed a nodular melanoma with a 1.47 mm Breslow index, with positive regression and rare mitoses. Ulceration and lymphovascular or perineural invasion were absent (Figure 3). A positive immunohistochemistry stain for Melan-A, SRY-related HMg-Box gene 10 (SOX-10), human melanoma black 45 (HMB-45), and Ki67 and negative for p16 confirmed the melanoma diagnosis (Figure 4).

**Figure 5:**
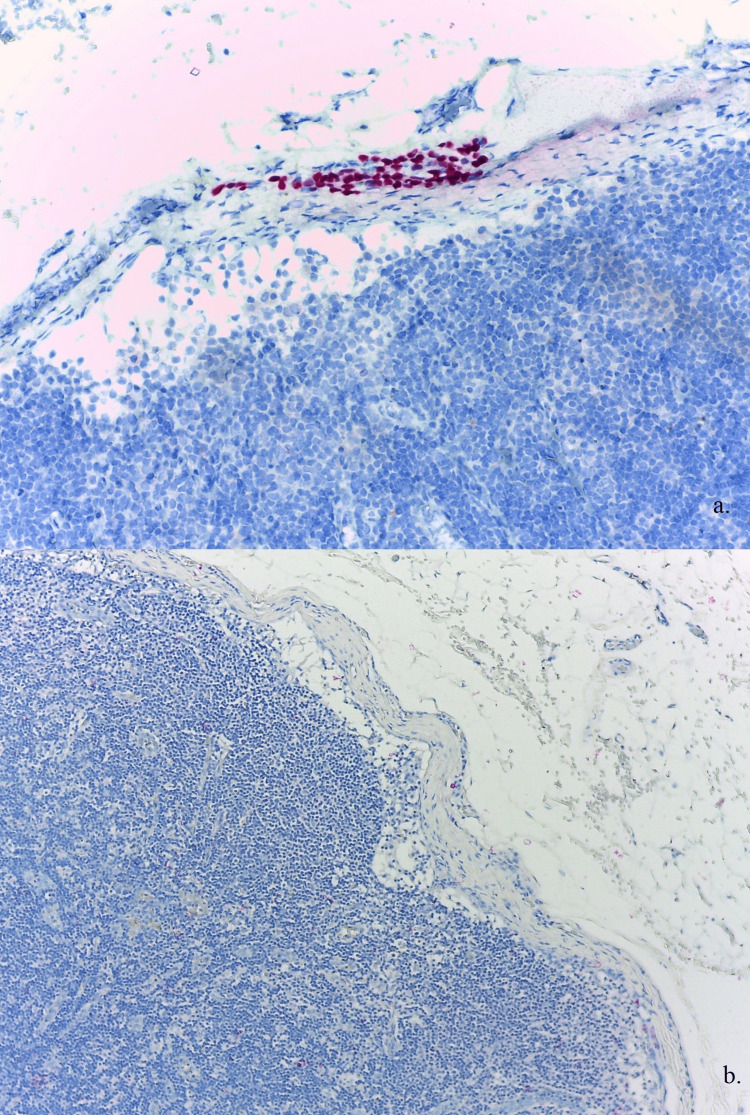
A cluster of melanocytes embedded within the lymph node capsule showing typical features of a nodal melanocytic nevus. a) Positive SOX-10 in nevus cells located in the subcapsular region of the lymph node (SOX-10 stain, original magnification: ×200). b) Nodal melanocytes negative for HMB-45; this can be used as an adjunct in the distinction between benign and malignant cells (HMB-45 stain, original magnification: ×100). SOX-10, SRY-related HMg-Box gene 10; HMB-45, human melanoma black 45

The patient underwent a sentinel lymph node biopsy (SLNB) of the right axillary lymph nodes. Regional lymph node metastases were absent at SLNB. However, a focus of melanocytic cells was present in two regional non-sentinel lymph nodes. These cells displayed a subcapsular distribution, positive for SOX-10 and negative staining for HMB-45. These features established the benign nature of the cells and a diagnosis of capsular nevus (Figure 5).

A full-body CT scan for distant metastases was negative, and this patient's melanoma was classified as pT2aN0M0 and stage IB according to the eighth edition of the American Joint Committee on Cancer (AJCC) staging system [1]. Screening for internal malignancies was negative. The genetic profiling (sensitivity: 98,85%; specificity: 98,9%) for high (*CDKN2A*, *BAP1*, and *CDK4*) and low and intermediate (*MITF*, *MLH1*, *MSH2*, *MSH6*, *NF1*, *PMS2*, *POT1*, *PTCH1*, *PTCH2*, *PTEN*, and *SUFU*) melanoma susceptibility genes was negative.

## Discussion

This paper reports a metachronous nodular melanoma of 1.4 mm Breslow thickness that arose two months after the follow-up visit and only one month after the COVID-19 infection, raising several questions regarding the potential immunosuppressive effect of SARS-CoV-2. The absence of both melanoma and blue-black dermoscopy criteria for nodular melanoma raised several questions regarding this case. The authors endorse excisional biopsy in patients with small erythematous nodules that display clinical and dermoscopy features of a non-melanocytic tumor, bearing in mind a disguising nodular melanoma.

Nodal nevi can be difficult to distinguish from nodal melanoma metastasis in the absence of a standardized method. A misdiagnosis of melanoma metastasis or nodal nevi leads to false staging and inadequate treatment. Intra-nodal nevi were previously described not only in patients with melanoma and with other malignancies, such as breast or prostate cancer, but also in patients without cancer [7-9]. Nodal melanocytic cells in the lymph nodes have been more frequently detected due to the increased use of SLNB. The prevalence of nodal nevi in melanoma patients reported in studies ranges from 3.9% to 25% [10].

There are two hypotheses regarding the origin of nodal nevi: a benign dissemination of cutaneous melanocytes or an embryonal migration from the neural crest [11,12]. The most frequent localization of the benign cells is in the capsule and trabeculae of the lymph nodes, while metastatic cells are located in the parenchyma [13]. The immunohistochemical melanocytic markers S-100, SOX-10, and Melan-A are sensitive for detection but cannot distinguish nodal nevus from metastatic melanoma. SRY-related HMg-Box gene 10 (SOX-10) is a key immunohistochemical marker for benign and malignant melanocytic tumors. It possesses high sensitivity (97%) for detecting metastatic melanoma in sentinel lymph nodes, in contrast with traditional markers such as S-100 (91%). HMB-45 displays a greater specificity but lacks sensitivity. Melan-A is less specific than SOX-10 and may lead to false-positive results [14-16]. The presence of intra-nodal nevi does not influence survival, and these melanoma patients are generally regarded as SLNB-negative [10,17].

Some studies have shown that SARS-CoV-2 alters human cell metabolism and increases glycolysis, thus enhancing tumor progression. On the other hand, some authors have described an oncolytic effect of the virus in lymphoma patients [18]. Leis et al. published three melanoma cases (amelanotic and hypopigmented) diagnosed after SARS-CoV-2 infection and hypothesized a potential tumorigenesis effect of the COVID-19 "cytokines storm" [19]. Similar to this patient, the tumors displayed either decreased or no pigmentation. A 2022 French cohort study showed that fast-growing melanomas usually present mutations of cell cycle pathway genes (*CDKNA* and *CDK4*) and receptor tyrosine kinase genes [20]. Still, this patient did not present any melanoma gene mutation. Consequently, the authors hypothesize that SARS-CoV-2 infection may have played a role in the progression of this fast-growing, atypical, nodular, malignant melanoma. Additionally, the Fitzpatrick II skin type and the atypical moles' syndrome were identified as risk factors for metachronous melanoma in this patient.

During the COVID-19 pandemic, patients' compliance dropped, and scheduled follow-up visits were postponed. These led to delayed diagnoses and a significant effect on life expectancy in skin cancer patients [6]. Hence, total body skin examination is mandatory during pandemic. Guidelines' recommendations regarding follow-up in melanoma patients should be applied.

## Conclusions

This clinical case posed many challenges, including clinical and dermoscopy diagnoses and lymph node assessment. Additionally, it demonstrates the importance of regular follow-up in melanoma patients, including during the pandemic. Studies regarding the carcinogenic or oncolytic effect of SARS-CoV-2 on various cell lines are warranted.
